# In Vitro Digestion and Fecal Fermentation of Low-Gluten Rice and Its Effect on the Gut Microbiota

**DOI:** 10.3390/foods12040855

**Published:** 2023-02-16

**Authors:** Zhi-Tao Li, Shuang-Xin Han, Jia-Yang Pu, Yu-Ying Wang, Yun Jiang, Min-Jie Gao, Xiao-Bei Zhan, Song Xu

**Affiliations:** 1 Key Laboratory of Carbohydrate Chemistry and Biotechnology, Ministry of Education, School of Biotechnology, Jiangnan University, Wuxi 214122, China; 2 State Key Laboratory of Food Science and Technology, Jiangnan University, Wuxi 214122, China; 3 Department of Hepatobiliary Surgery, Affiliated Hospital of Jiangnan University, Wuxi 214122, China

**Keywords:** low gluten, in vitro digestion, gut microbiota, short-chain fatty acids, probiotics

## Abstract

Low-gluten rice is part of a special diet for chronic kidney disease patients, but its digestive mechanism in the gastrointestinal tract is unclear. In this study, low-gluten rice (LGR), common rice (CR), and rice starch (RS) were used as experimental samples, and their digestion and bacterial fermentation were simulated using an in vitro gastrointestinal reactor to investigate the mechanism of the effect of LGR on human health. The starch digestibility of CR was higher than that of LGR, with statistically significant differences. LGR has growth-promoting and metabolic effects on *Akkermansia muciniphila.* Among the beneficial metabolites, the concentration of short-chain fatty acids (SCFAs) from LGR reached 104.85 mmol/L, an increase of 44.94% (versus RS) and 25.33% (versus CR). Moreover, the concentration of lactic acid reached 18.19 mmol/L, an increase of 60.55% (versus RS) and 25.28% (versus CR). Among the harmful metabolites, the concentration of branched-chain fatty acids (BCFAs) in LGR was 0.29 mmol/L and the concentration of ammonia was 2.60 mmol/L, which was 79.31% and 16.15% lower than CR, respectively. A significant increase in the concentration of the beneficial intestinal bacteria *Bacteroides* and *Bifidobacterium* occurred from LGR. The 16s rDNA sequencing showed that the abundance of the *Bacteroidetes* and *Firmicutes* increased and the abundance of the *Proteobacteria* and *Fusobacteria* decreased. Thus, LGR has positive effects on digestion and gut microbiota structure and metabolism in humans.

## 1. Introduction

Chronic kidney disease is a major health hazard for human beings, with at least 850 million people suffering from kidney disease worldwide, including more than 400 million in Asia [1]. A low gluten diet is a staple for people with chronic kidney disease. This diet benefits people with chronic kidney disease as it delays the progression of kidney disease and improves intestinal function. Hansen et al. [2] found that a low gluten diet induced moderate changes in the gut microbiome, reduced fasting and postprandial hydrogen exhalation, and improved abdominal distension. Rice is one of the main diets of Asians. The relative content of protein in rice is approximately 8% to 10% and can be divided into albumin, globulin, alcoholic protein, and glutenin according to their solubility [3]. Gluten is the main protein storage in rice seeds and the main soluble protein. Moreover, gluten is rich in lysine which is easily digested and absorbed by the human body, is located in protein body II inside the endosperm cells, and accounts for approximately 80% of the total protein dry mass of rice [4,5]. However, excessive absorption of soluble protein may lead to disorders of protein metabolism, especially for patients with kidney disease, who should not consume rice with a soluble protein amount fraction of more than 4% [6,7]. Low-gluten rice (LGR) may provide potential health benefits for patients with chronic kidney disease, but only few studies have examined the effects of LGR on the structure and metabolism of gut microbiota in the large intestine after digestion through the stomach and small intestine.

Inhabited by a highly diverse microbial community composed mainly of bacteria, the human gut is the largest and most complex micro-ecosystem in the human body [8,9]. In addition to absorbing nutrients and excreting waste, the microbiota present in the human gastrointestinal tract can also produce beneficial or harmful substances which can affect human health, such as short-chain fatty acids (SCFAs) and branched-chain fatty acids (BCFAs). *Bifidobacterium* and *Lactobacillus* synthesize vitamins required by the body, promote the production of SCFAs and inhibit the production of BCFAs. Among SCFAs acetic acid promotes the development of peripheral tissues, and propionic acid stimulates hepatocyte growth [10]. Butyric acid stimulates the growth of intestinal epithelial tissues and promotes normal maturation and differentiation of intestinal cells [11]. However, BCFAs are irritating to the mucosa and may trigger inflammation [12,13]. *Akkermansia muciniphila* is valuable in improving host metabolic function and immune response by affecting metabolism through enhancing the integrity of the intestinal wall, thereby reducing intestinal permeability and associated endotoxemia [14]. *Fecal coliform* can cause a variety of local tissue and organ infections in humans and animals under certain conditions. Clearly, gut microbiota is an important player in human metabolism as it can (1) provide beneficial or harmful substances, enzymes, and energy to human metabolism, (2) help clarify intestinal function and hidden metabolic or immune disease problems, as gut microbiota are important participants in human metabolism, and (3) provide multifaceted probing data for medical diagnosis [15]. Therefore, studying the beneficial effects of LGR on gut microbiota would be beneficial.

The use of in vivo digestion to examine the digestive process of food and drugs in the human body often has the disadvantages of high cost, long experimental period, and poor reliability [16]. Thus, the trend has gradually evolved toward that of in vitro digestion to simulate the human intestine. In vitro digestion is divided into static and dynamic simulations. Static simulation cannot model the physical processes such as shearing and mixing that occur in vivo and does not absorb the metabolites of the digestion process. Dynamic simulation compensates for the shortcomings of static simulation by not only being able to simulate physical processes in the gastrointestinal tract but also allowing for the observation of other changes that occur at different digestion times [17].

In this study, LGR, common rice (CR), and rice starch (RS) were used as experimental samples to investigate the digestion kinetics of LGR in the stomach and small intestine and the mechanism of influence on intestinal gut microbiota using the developed dynamic simulation equipment, namely the bionic gastrointestinal reactor (BGR) [18] and bionic colon model (BCM) [19], to elucidate the mechanism of influence of a low-gluten diet on the host for reference.

## 2. Materials and Methods

### 2.1. Experimental Materials

LGR was provided by the Institute of Crop Science, the Chinese Academy of Agricultural Sciences, Beijing, China; CR was purchased from supermarkets; and other reagents were purchased from the Sinopharm Chemical Reagent Co., Shanghai, China.

### 2.2. In Vitro Gastric and Small Intestine Digestion

The digestion process was modified from the previous method by referring to Li et al. [20]. The prepared rice was added to the BGR (Supplementary Figure S1) by feeding peristaltic pump flow, along with saliva, gastric juice, and intestinal fluid as described in the literature. Extractions with trichloroacetic acid (TCA) and subsequently quantified the nitrogenous compounds in the TCA soluble extract to study the extent of starch breakdown and protein hydrolysis in the simulated chyme. Digests were treated rapidly with TCA at 4 °C to stop all enzymatic hydrolysis reactions and to separate small peptides, amino acids and sugars from high molecular weight proteins and enzymes. Five (5) mL of minced food was treated with 700 μL of 100% TCA (12% final TCA concentration) in a centrifuge tube and the mixture was vortexed. After incubation at 4 °C for 1 h, the sample was centrifuged at 14,000× *g* for 45 min at 4 °C. The supernatant was aspirated, divided into aliquots and stored at −20 °C prior to analysis. Monosaccharides were applied enzymatically with glucose oxidase and measured colorimetrically, protein hydrolysis was determined by micro Kjeldahl method by equations referring to C. Villemejane et al. [21].

### 2.3. Culture of Gut Microbiota in the Colon In Vitro

The fermentation process was performed according to Li et al. [19]. At 37 °C, *A. muciniphila*, *Bifidobacterium*, *Lactobacillus, Bacteroid,* fecal coliform, total anaerobic bacteria, and fecal were cultured using selection media (Supplementary Table S1). The OD_600_ value of bacterial solution was determined by ultraviolet spectrophotometer (Nanodrop 2000, Wilmington, DE, USA) and the MRS liquid culture medium was used as a reference to determine an initial OD_600_ value, and then the culture was continued. Informed consent was obtained from all participants, as required by the Human Ethics Committee of Affiliated Hospital of Jiangnan University (approval No. JY-0016) on the Use of Humans as Experimental Subjects. The feces of three selected volunteers were mixed well and inoculated into the BCM, and after 24 h of colonization, the digested RS, CR, and LGR were added to the BCM for fermentation.

### 2.4. SCFAs, BCFAs, and Lactic Acid

SCFAs, BCFAs, and lactic acid were extracted from the samples using ether and ascertained with the Agilent (7820 A, Palo Alto, CA, USA) gas chromatograph following the method of Wang et al. [22]. The SCFAs and BCFAs were separated using a HP-INNOWAX (19091N-133, Billerica, MA, USA) capillary column with an inner diameter of 250 μm and a film thickness of 0.25 μm. The chromatographic ramp-up procedure was as follows: an initial temperature of 60 °C was maintained for 1 min, ramped up to 190 °C at 20 °C/min and maintained for 7.5 min; the injection port temperature was 220 °C; the hydrogen flame ionization detector temperature was 250 °C; the injection volume was 5 μL; the shunt ratio was 20:1; the carrier gas was high-purity nitrogen (purity > 99.999%) at a flow rate of 1.5 mL/min; the tail gas was high-purity nitrogen (purity > 99.999%) at a flow rate of 30 mL/min; the hydrogen flow rate was 40 mL/min; and the air flow rate was 400 mL/min.

### 2.5. Determination of the Ammonia Content

The ammonia content in the fermentation broth was determined using the indophenol blue-spectrophotometric method. First, Solution A was obtained by adding 5 g of phenol and 2.0 mL of sodium nitrosoferricyanide solution (1.25%) to 400 mL of water and fixing the volume to 500 mL. Then, Solution B was obtained by adding 2.5 g of NaOH, 3.5 mL of NaClO, and 2.0 g of trisodium citrate to 400 mL of water and fixing the volume to 500 mL. Subsequently, two fermentation broth samples of 100 μL each were singly introduced to 5 mL of Solution A and Solution B, mixed thoroughly, and stored at 37 °C. The resulting solutions were placed in a water bath at 37 °C for 20 min, removed to cool to room temperature (Wuxi, 20 °C), and measured for absorbance at 637 nm in the Bio-rad (Hercules, CA, USA).

### 2.6. S rDNA Amplicon Sequencing Method

The DNA extracted from the fermentation broth was diluted to 1 ng/μL, and the 16S V3–V4 region was PCR amplified using the specific primers with Barcode as the template for the diluted genomic DNA. The obtained PCR products were then purified, and library construction was performed by using the TruSeq^®^ DNA PCR-Free Sample Preparation Kit. The constructed library was quantified using Qubit and Q-PCR and subsequently sequenced on the machine using NovaSeq6000. Based on the characteristics of the amplified 16S region, a small fragment library was constructed, and the library was sequenced based using the Illumina NovaSeq (San Diego, CA, USA) sequencing platform. The sequencing was entrusted to the Beijing NovaSeq Technology Co.

### 2.7. Statistical Analysis

All experiments were repeated three times and averaged, and ANOVA was performed using the F-test of the software SPSS (Version 26.0, Statistical Product Service Solutions), and the Duncan test was used to analyze the significance of differences, with *p* < 0.05 being considered significant. Graphpad Prism (Version 8.4.3; Graphpad Software) was used for graphing.

## 3. Results and Discussion

### 3.1. Starch Digestibility

**Figure 1:**
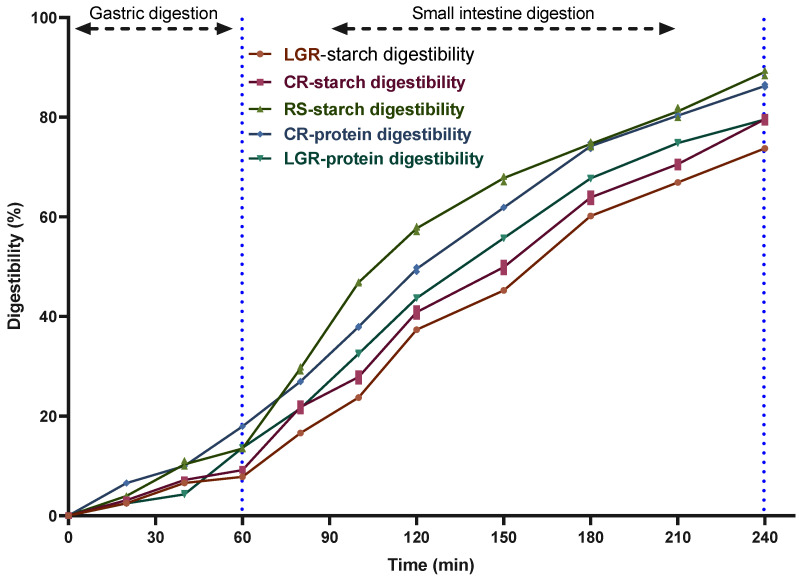
Starch and protein digestibility of LGR, CR, and RS in dynamic digestion simulation.

Figure 1 shows the starch and protein digestibility of LGR, CR and RS as measured using the BGR. The starch and protein digestibility of CR was higher than that of LGR with a significant difference (*p* < 0.05), probably because of the cross-linking of insoluble proteins in LGR with starch, a feature that affects starch digestibility [23]. Starch-binding proteins are often referred to as starch granule-associated proteins, and they form a protective network around the starch granules, making them less susceptible to digestion by digestive enzymes. Consistent with the results from Amina et al. [24] which indicates that removal of proteins increases starch digestibility of rice, CR has high gluten content, making it a soluble protein which is easily hydrolyzed, but LGR has low gluten content, is a highly alcoholic soluble protein, has a dense structure, and is not easily hydrolyzed. Low starch digestibility results in a lower rate of conversion of starch to sugar, making the intake of LGR healthier for some people who need to strictly limit their sugar and protein intake.

### 3.2. A. muciniphila Growth and Metabolism

**Figure 2:**
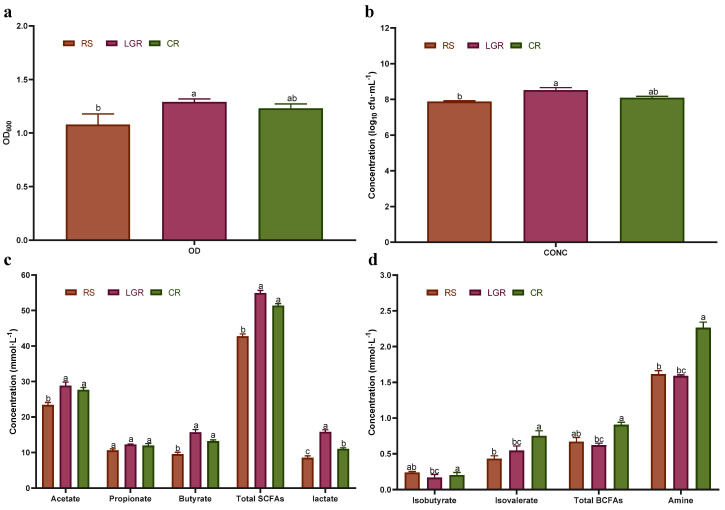
Effect of LGR, CR, and RS on *Akkermansia muciniphila* growth and metabolism in dynamic fermentation simulation. Note: OD_600_ (**a**), growth concentration (**b**), changes in SCFAs (**c**), changes in BCFAs (**d**). The data are shown as the mean ± SD (*n* = 3) and analyzed using one−way ANOVA with Tukey’s test, differences marked with the same lowercase letter in the graph indicate that the differences are not significant (*p* > 0.05), different lowercase letters indicate significant differences (*p* < 0.05); LGR: low gluten rice; CR: common rice; RS: rice starch.

To investigate the in vivo effect of LGR on *A. muciniphila*, the BCM was employed to ferment *A. muciniphila* using RS, CR, and LGR as nutritional composition. The changes in colony concentration and metabolite content were analyzed to assess the effect of LGR on *A. muciniphila* growth and metabolism. As shown in Figure 2a,b, the OD600 and colony concentration of *A. muciniphila* with LGR as carbon source were higher than those with RS and CR, with significant differences (*p* < 0.05). Compared with RS, LGR had a 24.75% increase in OD600 and 0.58 log10 cfu/mL increase in colony concentration. Thus, LGR could promote *A. muciniphila* growth. Zhang et al. [14] found that *A. muciniphila* was associated with many metabolic diseases and had a preventive effect on obesity and type 2 diabetes, as well as reducing the risk of cardiovascular disease, with the SCFAs produced by its metabolism being one of the main factors of action [25]. As shown in Figure 2c, the concentration of total SCFAs in LGR reached 54.33 mmol/L, which was 28.56% higher than that in RS; the concentration of acetic acid reached 28.09 mmol/L, which was 22.61% higher than that in RS; the concentration of propionic acid reached 26.68 mmol/L, which was not significantly different from that in RS and CR (*p* > 0.05). The concentration of butyric acid reached 15.15 mmol/L in LGR, which was 65.57% and 23.67% higher than that in RS and CR, respectively. The concentration of lactic acid was also elevated in LGR at 15.4 mmol/L, which was 89.42% and 42.59% higher than that with RS and CR. The present results suggest that LGR not only promotes *A. muciniphila* growth but also stimulates the production of beneficial SCFAs and lactate metabolism by *A. muciniphila*. By contrast, BCFAs metabolized by gut microbiota may have negative effects on human health [26]. As shown in Figure 2d, no significant difference was noted between the isobutyric and isovaleric acid content in LGR compared with those in CR, and the concentration of total BCFAs in LGR was 0.64 mmol/L, which was 37.50% lower than that in CR. The concentration of ammonia in LGR was also 2.36 mmol/L, which was 23.31% lower than that in CR. Comparisons with RS revealed no significant differences. *A. muciniphila* may prefer to feed on alcohol-soluble proteins, and LGR is rich in alcohol-soluble proteins, which can better promote *A. muciniphila* growth and produce more short-chain fatty acids such as acetate and propionic acid during metabolism, and inhibit the production of branched-chain fatty acids through competitive rejection, making it beneficial to human health.

### 3.3. Gut Microbiota Growth and Metabolism

**Figure 3:**
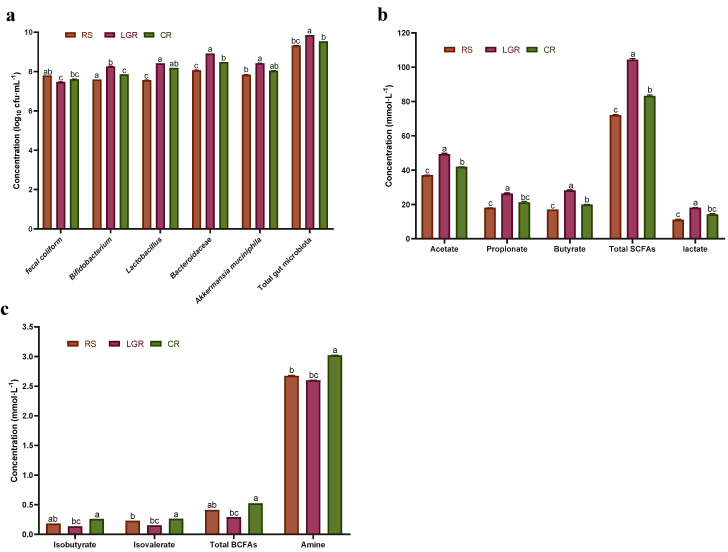
Effect of LGR, CR, and RS on gut microbiota growth and metabolism in dynamic fermentation simulation. Note: changes in microbiota (**a**), changes in SCFAs (**b**), changes in BCFAs and amine (**c**). The data are shown as the mean ± SD (*n* = 3) and analyzed using one−way ANOVA with Tukey’s test, differences marked with the same lowercase letter in the graph indicate that the differences are not significant (*p* > 0.05), different lowercase letters indicate significant differences (*p* < 0.05); LGR: low gluten rice; CR: common rice; RS: rice starch.

To investigate LGR-induced in vivo changes of microbial growth and metabolism in the colon, the fermentation of feces using the BCM with RS, CR, and LGR as nutritional composition were used to analyze the changes of microbial concentration and metabolite content and assess the effects of LGR on the growth and metabolism of gut microbiota. The results of the partial gut microbiota counting using the plate counting method are shown in Figure 3a. The concentration of intestinal beneficial bacteria in LGR increased compared with that in RS and CR, with the most significant increase in *Bifidobacterium* and *Bacteroides*, with concentrations of 8.27 log10 cfu/mL and 8.92 log10 cfu/mL. In terms of enteropathogenic bacteria, the fecal *E. coli* concentration in LGR was 7.39 log10 cfu/mL. Thus, LGR could increase the concentration of beneficial intestinal bacteria and decrease the concentration of harmful intestinal bacteria in healthy people.

In terms of beneficial metabolites, Figure 3b shows that compared with RS and CR, LGR reached a concentration of 49.70 mmol/L of acetic acid, which increased by 33.60% and 18.42%, respectively; of 26.68 mmol/L of propionic acid, which increased by 47.32% compared with RS; of 28.47 mmol/L of butyric acid, which increased by 67.18% and 41.78%, relative to RS and CR, respectively; and the total SCFAs concentration of LGR reached 104.85 mmol/L, which was 44.94% and 25.33% higher than that of RS and CR, respectively. The lactate concentration was also significantly higher in LGR compared to RS, reaching 18.19 mmol/L, an increase of 60.55%. The high content of soluble gluten in CR shifted the microbiota to protein fermentation, while the low content of gluten in LGR shifted the microbiota to carbohydrate fermentation, with SCFAs and lactate as the predominant metabolites. Thus, LGR has a significant facilitative effect on the metabolism of SCFAs and lactate by the gut microbiota.

In terms of harmful metabolites, Figure 3c shows that the concentrations of isobutyric acid, isovaleric acid, total BCFAs, and ammonia were significantly reduced in LGR, where the concentration of isobutyric acid was 0.14 mmol/L, which was 85.71% lower than that of RS; the concentration of isovaleric acid was 0.15 mmol/L, which was 73.33% lower than that of CR; the concentration of total BCFAs was 0.29 mmol/L which was 79.31% lower than that of RS; and the concentration of ammonia was 2.60 mmol/L, which was 16.15% lower than that of CR. The high content of soluble gluten in CR allows the microbiota to shift to protein fermentation [3], but the low gluten content in LGR means relatively more fermentable carbohydrates are the main substance.

### 3.4. Gut Microbiota Structure

**Figure 4:**
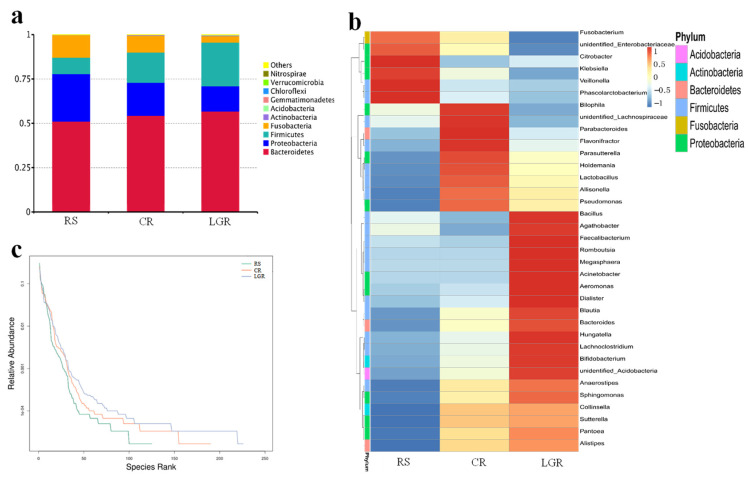
Effect of LGR, CR, and RS on gut microbiota composition in dynamic fermentation simulation. Note: changes in phylum level (**a**), changes in genus level (**b**), changes in species diversity curve (**c**). LGR: low gluten rice; CR: common rice; RS: rice starch.

As shown in Figure 4a, the dominant microbiota at the phylum level consisted of *Bacteroidetes, Firmicutes, Proteobacteria*, and *Fusobacteria*, accounting for more than 95% of the total microbiota. Compared to RS, *Bacteroidetes* and *Firmicutes* increased and *Proteobacteria* and *Fusobacteria* decreased in CR and LGR, with the change being particularly pronounced in LGR. This outcome indicates that *Bacteroidetes* and *Firmicutes* have a mutually reinforcing symbiotic relationship, and they jointly promote host energy absorption and storage [27]. Moreover, *Proteobacteria* and *Fusobacteria* are typical markers of gut microbiota dysbiosis [28], and the significant decrease in the number of *Proteobacteria* in LGR may be caused by the increased production of SCFAs and lactic acid, which, in turn, lowered the intestinal pH and inhibited the growth of harmful intestinal bacteria. Therefore, LGR could improve the structure of gut microbiota at the genus level in healthy people and promote the ecological balance of gut microbiota. As shown in Figure 4b, the composition of the gut microbiota varied considerably at the genus level. Compared to the RS and CR counterparts, the relative abundance of *Bilophila, Parabacteroides*, and *Flavonifracto* were significantly higher. *Bilophila* can cause primary septic infections and severe secondary infections [29], *Parabacteroides* can produce beneficial metabolic end products such as acetic acid and succinic acid [30], and *Flavonifracto* consist mostly of conditionally pathogenic bacteria [31]. The relative abundance of *Fusobacterium*, *Citrobacter*, and *Klebsiella*, which are pathogenic [32,33,34], were significantly reduced in the LGR, as consistent with the results of Bonder et al. [35] who found that low gluten grains reduced the abundance of *Fusobacterium*. The relative abundance of *Fusobacterium* in the LGR. The relative abundance of *Bacillus*, *Faecalibacterium*, *Romboutsia*, and *Megasphaera* were significantly increased. Among these, *Bacillus* could antagonize harmful bacteria in the intestine, *Faecalibacterium* was the main genus of butyric acid-producing bacteria [36], *Romboutsia* had an important role in maintaining the health status of the host [37], and *Megasphaera* was involved in the fermentation of lactic acid and fructose in humans [38]. In addition, LGR can also effectively increase the richness and uniformity of gut microbiota (Figure 4c). Taken together, these results suggest that LGR can improve the structure of the host gut microbiota and promote the increase in the abundance of beneficial bacterial genera.

## 4. Conclusions

The results of this study showed that starch and protein digestibility of CR was higher than that of LGR during in vitro gastric and small intestinal digestion. LGR promotes the metabolic production and probiotic growth of SCFAS and lactic acid, and inhibits the metabolic production and harmful bacterial growth of BCFAs and ammonia. However, excessive intake of LGR may also stimulate disproportionate growth of certain bacteria or pathogens, which may endanger intestinal homeostasis. As far as the current results are concerned, further in vivo digestion experimental studies in animals are needed to obtain more detailed gut microbiota regulatory mechanisms, which will lay a solid foundation for the mechanism of LGR for human health promotion.

## Data Availability

The data presented in this study are available on request from the corresponding author. The data are not publicly available due to experimental restrictions.

## Supplementary Materials

The following supporting information can be downloaded at: https://www.mdpi.com/article/10.3390/foods12040855/s1, Figure S1: dynamic simulation equipment; Table S1: Components of selective media.
