# Supplementary material for: In Vitro Digestion and Fecal Fermentation of Low-Gluten Rice and Its Effect on the Gut Microbiota
Source: Foods. 2023 Feb 16;12(4):855. doi: 10.3390/foods12040855 (PMC9956666; doi:10.3390/foods12040855)
Supplement: Supplementary file 1 [file foods-12-00855-s001.zip › foods-2126593-supplementary.pdf]

**Table S1.** Components of selective media

| Microbiota | Culture medium (g/L) |
|---|---|
| *Faecal coliform* | Tryptone 20.0, lactose 10.0, bovine bile salt 5.0, NaCl 5.0, neutral red 0.075, agar 20.0 |
| *Lactobacillus* | Tryptone 10.0, beef powder 10.0, yeast powder 5.0, glucose 20.0, Tween-80 1.0, K2HPO4 2.0, sodium acetate 5.0, trisodium citrate 2.0, magnesium sulfate 0.1, manganese sulfate 0.05, L-cysteine 0.5, agar 20.0 |
| *Bacteroid* | Tryptone 20.0, yeast powder 5.0, soluble starch 0.5, xylan 5, NaCl 5.0, K2HPO4 0.05, KH2PO4 0.05, L-cysteine hydrochloride 1.0, heme chloride 0.01, vitamin K1 0.002, kanamycin 0.1, vancomycin 0.0075, bromocresol violet 0.012, agar 20.0 |
| *Bifidobacterium* | Tryptone 15.0, dextrose 20.0, yeast powder 2.0, soluble starch 0.5, NaCl 5.0, liver extract 5.0, Tween-80 1.0, mupirocin 0.05, agar 20.0, tryptone 5.0, casein peptone 16.0, bovine brain extract 4.0, bovine heart extract 4.0 |
| Total anaerobic bacteria | Glucose 2.0, NaCl 5.0, Na2HPO4 2.5, agar 13.5 |
| Fecal | Starch 6.0, arabinogalactan 1.0, pectin 2.0, xylan 1.0, glucose 0.4, yeast powder 3.0, L-cysteine 0.5, mucin 2.0, KCl 1.0, NaCl 4.5, K2HPO4 0.5, KH2PO4 0.5, CaCl2-6H2O 0.15, MgSO4-7H2O 0.01, FeSO4-7H2O 0.005, heme chloride 0.025, Tween-80 1.0, bile salts 0.4, vitamin mixture 0.01, Menaquinone 1.0, D-biotin 2.0, pantothenic acid 10.0, nicotinamide 5.0, p-aminobenzoic acid 5.0 |
| *Akkermansia muciniphila* | Tryptone 5.0, casein peptone 16.0, bovine brain extract 4.0, bovine heart extract 4.0 Glucose 2.0, NaCl 5.0, Na2HPO4 2.5, mucin 2.0 |

Note: The above medium was supplemented with low-gluten rice (LGR), common rice (CR), and rice starch (RS) all at 2 g/L.

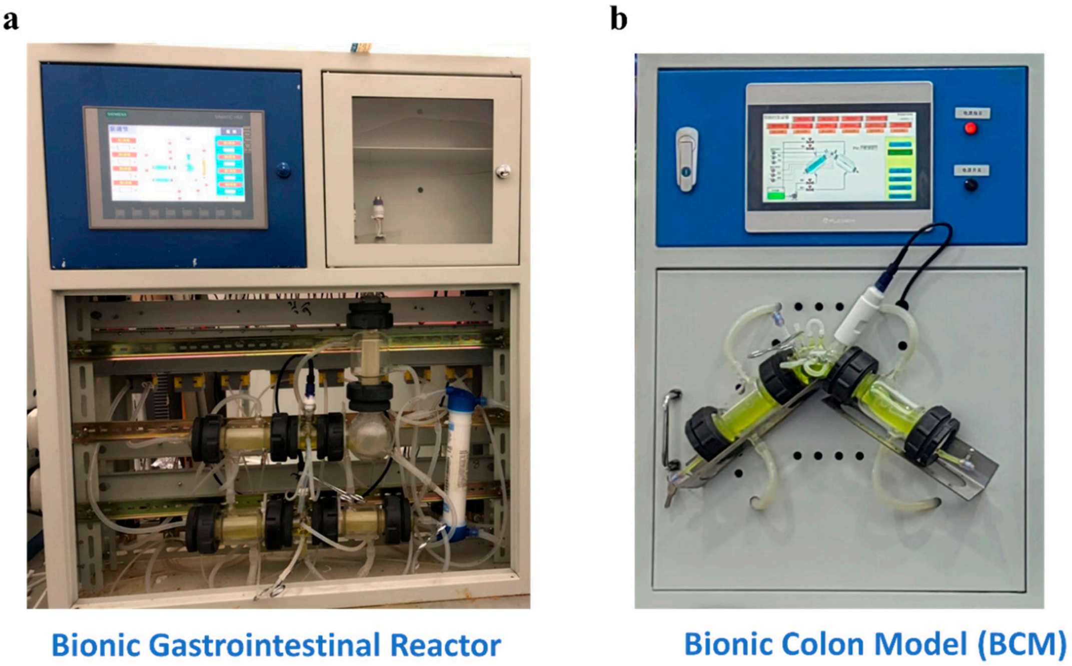


**Figure S1.** Dynamic simulation equipment. (**a**) bionic gastrointestinal reactor (BGR); (**b**) bionic colon model (BCM).
